# Correction to “Running economy and lower‐limb anthropometry in adult male Kenyan and Danish middle‐ and long‐distance runners and in untrained adolescents”

**DOI:** 10.1113/EP094364

**Published:** 2026-09-19

Larsen, H. B., Boit, M. K., Adamsen, M. L., Madsen, F., Berg, R. M. G., Hanel, B, and Søndergaard, H. (2025). Running economy and lower‐limb anthropometry in adult male Kenyan and Danish middle‐ and long‐distance runners and in untrained adolescents. *Experimental Physiology*, *111*(4), 1919–1932. https://doi.org/10.1113/EP092758

This article corrects some errors in the originally published article.

The author Hans Søndergaard has been added after being mistakenly omitted originally. He contributed to study conception, coordination, data acquisition and interpretation. Due to this omission, he did not review the manuscript prior to publication but has subsequently reviewed and approved the published article and agrees to be accountable for his contributions to the work.

Anthropometric and running economy data for some of the Kenyan adolescents have previously been reported in studies addressing separate research questions, comparing Nandi town and village boys with respect to physical activity and physiological characteristics, and their responses to endurance training, respectively (Larsen et al., 2004, 2005). These publications should have been cited in the original article.

In the abstract and main text, the number of Kenyan sub‐elite runners is incorrectly stated as 29. The correct number is 38, as stated in Tables 1 and 2, and Table A1 in the Appendix. The number of Kenyan untrained adolescents is incorrectly stated as 46. The correct number is 24, as stated in Tables 1 and 2, and Table A1 in the Appendix.

**TABLE 1B:** Participant characteristics for Danish and Kenyan male adolescents.

	Denmark	Kenya		
	Untrained (*N* = 30)	Untrained (*N* = 24)	Junior runner (*N* = 6)	*P*‐value ANOVA
**Age (Years)**				
Mean (SD)	16.5 (1.4)	16.6 (0.64)	16.1 (1.7)	0.699
**Height (cm)**				
Mean (SD)	176 (7.8)	170 (7.2)	169 (7.6)	0.00345
**Weight (kg)**				
Mean (SD)	65.1 (10.8)	53.0 (6.2)	53.7 (10.1)	<0.001
**BMI (kg∙m^−^^2^)**				
Mean (SD)	20.8 (2.3)	18.4 (1.6)	18.6 (1.9)	<0.001

**TABLE 2:** Lower‐limb anthropometry. A: Adult male Danish and Kenyan middle‐ to long‐distance runners. B: Danish and Kenyan male adolescents.

A	Denmark		Kenya		
	Subelite (*N* = 37)	Elite (*N* = 20)	Subelite (*N* = 38)	Elite (*N* = 12)	*P*‐value ANOVA
**Total Leg Length (cm)**					
Mean (SD)	103 (5.8)	104 (4.8)	102 (4.3)	102 (4.3)	0.698
Missing	19 (51.4%)	10 (50.0%)	5 (13.2%)	6 (50.0%)	
**Relative Leg Length (%)**					
Mean (SD)	57.0 (1.2)	57.5 (1.0)	59.5 (1.2)	59.4 (0.5)	<0.001
Missing	19 (51.4%)	10 (50.0%)	5 (13.2%)	6 (50.0%)	
**Lower‐Leg Length (cm)**					
Mean (SD)	43.8 (2.5)	44.7 (2.4)	45.1 (2.2)	45.9 (2.0)	0.159
Missing	19 (51.4%)	10 (50.0%)	5 (13.2%)	6 (50.0%)	
**Relative Lower‐Leg Length (%)**					
Mean (SD)	42.6 (0.9)	42.9 (1.0)	44.2 (0.7)	44.9 (0.8)	<0.001
Missing	19 (51.4%)	10 (50.0%)	5 (13.2%)	6 (50.0%)	
**Lower‐Leg Volume (cm^3^)**					
Mean (SD)	3500 (486)	3380 (452)	3000 (451)	2870 (356)	<0.001
Missing	19 (51.4%)	10 (50.0%)	5 (13.2%)	6 (50.0%)	
**Calf Volume (cm^3^)**					
Mean (SD)	2670 (409)	2580 (347)	2180 (338)	2120 (287)	0.00155
Missing	20 (54.1%)	10 (50.0%)	26 (68.4%)	7 (58.3%)	
**Foot Volume (cm^3^)**					
Mean (SD)	816 (103)	793 (113)	816 (124)	807 (110)	0.957
Missing	20 (54.1%)	10 (50.0%)	26 (68.4%)	7 (58.3%)	

B	Denmark	Kenya		
	Untrained (*N* = 30)	Untrained (*N* = 24)	Junior runner (*N* = 6)	*P*‐value ANOVA
**Total Leg Length (cm)**				
Mean (SD)	100 (5.9)	102 (5.1)	102 (5.6)	0.535
Missing	0 (0%)	0 (0%)	0 (0%)	
**Relative Leg Length (%)**				
Mean (SD)	56.7 (1.3)	59.9 (2.1)	60.2 (1.8)	<0.001
Missing	0 (0%)	0 (0%)	0 (0%)	
**Lower‐Leg Length (cm)**				
Mean (SD)	44.2 (2.5)	45.8 (3.0)	45.0 (2.7)	0.0951
Missing	0 (0%)	0 (0%)	0 (0%)	
**Relative Lower‐Leg Length (%)**				
Mean (SD)	44.1 (1.0)	45.1 (1.8)	44.2 (0.9)	0.0431
Missing	0 (0%)	0 (0%)	0 (0%)	
**Lower‐Leg Volume (cm^3^)**				
Mean (SD)	3490 (466)	2270 (376)	NA (NA)	<0.001
Missing	0 (0%)	12 (50.0%)	6 (100%)	
**Calf Volume (cm^3^)**				
Mean (SD)	2610 (395)	1850 (410)	NA (NA)	<0.001
Missing	0 (0%)	15 (62.5%)	6 (100%)	
**Foot Volume (cm^3^)**				
Mean (SD)	882 (102)	493 (198)	750 (125)	<0.001
Missing	0 (0%)	15 (62.5%)	0 (0%)	

The method used to calculate mean cross‐sectional area of the lower leg (mean lower‐leg thickness) was not described fully. The authors wish to clarify that this variable was calculated as lower‐leg volume (including foot and calf volumes) divided by lower‐leg length.

Figure 4B was incorrectly interpreted in the text of the original article. In section 3.4 of the Results, the text reads: “In untrained adolescents, the volume of section 2 was lower in Kenyans than in Danes”. The text should read: “In untrained adolescents, the volume of section 2 was lower in Danes than in Kenyans”.

Related to this, the text in the Discussion incorrectly reads: “In contrast, sections 1 and 2 (i.e. the distal and lower‐mid sections of the calf) were slimmer in untrained Kenyan adolescents, a pattern for which we currently have no clear explanation, but the difference from adult runners might somehow reflect adaptations to years of exercise training by running.” This should instead read: In contrast, the volume of section 2 (i.e. the lower‐mid section of the calf) was slightly higher in untrained Kenyan adolescents than in Danes, a pattern for which we currently have no clear explanation.″

This article also corrects some issues relating to Tables 1 and 2. A formatting misalignment between Parts A and B may incorrectly suggest that two adjacent columns represented Danish untrained participants. The right‐hand column relates to Kenyan participants. In Table 2A, relative leg length for Danish elite runners was reported incorrectly as: 52.2 (0)% with an ANOVA *P* = 0.03. It should read: 57.7 (1.0)% with an ANOVA *P* < 0.001. In Table 2A, several lower‐leg and calf‐volume values for Kenyan elite runners were incorrectly classified, resulting in lower‐leg volume being reported in place of calf volume and omission of calf‐volume values. These values can be seen in the corrected Table 2A, which is provided alongside corrected version of Tables 1B and 2B below.

Following identification of these errors, all relevant analyses were rerun using the corrected dataset. Calf‐volume data are now correctly reported for Kenyan elite runners, permitting direct comparisons with the other groups, and as expected, showing that calf volume was lower in Kenyan elite runners. The mean lower‐leg volume for Kenyan elite runners, previously reported as 2200 (SD 326) cm^3^, is corrected to 2870 (SD 356) cm^3^. While a reduction in lower‐leg volume relative to the other groups remains, it is thus less pronounced than previously reported. The ANOVA p‐value for calf volume changes from 0.0037 to 0.00155. All other p‐values remain unchanged.

Apart from the changes described above, the effect sizes and statistical results are materially unchanged. The statistical inferences and conclusions of the article are unchanged.

We apologise for these errors.
